# Major Aortic Reconstruction with the Replacement of Supra-Aortic Branches: Successful Surgical Treatment of Takayasu Arteritis Initially Presented as Congestive Heart Failure

**DOI:** 10.3390/diagnostics11020216

**Published:** 2021-02-02

**Authors:** Maja Stojanovic, Sanvila Raskovic, Marija Boricic-Kostic, Vesna Bozic, Maja Vuckovic, Aleksandra Peric-Popadic, Ilija Bilbija

**Affiliations:** 1 Clinic for Allergy and Immunology, Clinical Center of Serbia, Faculty of Medicine, University of Belgrade, 11000 Belgrade, Serbia; sanvilar28@yahoo.com (S.R.); ppaleksandra@yahoo.com (A.P.-P.); 2 Clinic for Cardiology, Clinical Center of Serbia, 11000 Belgrade, Serbia; marijaboricickostic@yahoo.com; 3 Department of Pathology, Clinic for Cardiac Surgery, Clinical Center of Serbia, 11000 Belgrade, Serbia; vesna.bozic.ph@gmail.com; 4 Department of Radiology and Magnetic Resonance, Clinical Center of Serbia, 11000 Belgrade, Serbia; majaimaging@gmail.com; 5 Clinic for Cardiac Surgery, Clinical Center of Serbia, Faculty of Medicine, University of Belgrade, 11000 Belgrade, Serbia; i.bilbija@yahoo.com

**Keywords:** ultrasound, inflammation, vascular–endovascular surgery, Takayasu arteritis, aortic disease, vasculitis

## Abstract

Takayasu arteritis (TA) is a rare, large vessel vasculitis that affects aorta, its major branches, and occasionally pulmonary arteries. Patients with TA can present with constitutional features and/or various symptoms and signs caused by morphological changes in the blood vessels affected by the inflammatory process. Corticosteroids (CS) and immunosuppressives (IS) are the first line treatment for active TA. Open surgery remains a treatment of choice for TA patients with moderate-to-severe aortic regurgitation (AR) and ascending aortic aneurysm (AAA). We present a 26-year-old female diagnosed with an advanced stage of TA, initially presented as congestive heart failure. Due to a progressive course of the disease (AR 3+, AAA 5.5 cm), surgery of the Aortic valve and root (Bentall procedure), with total arch reconstruction and replacement of supra-aortic branches was performed. The patient has had an uneventful recovery during the postoperative course with no complications at one year follow-up. Normal left ventricle (LV) diameter, LV ejection fraction 67%, and a trace of AR were seen on the last echocardiography.

**Figure 1:**
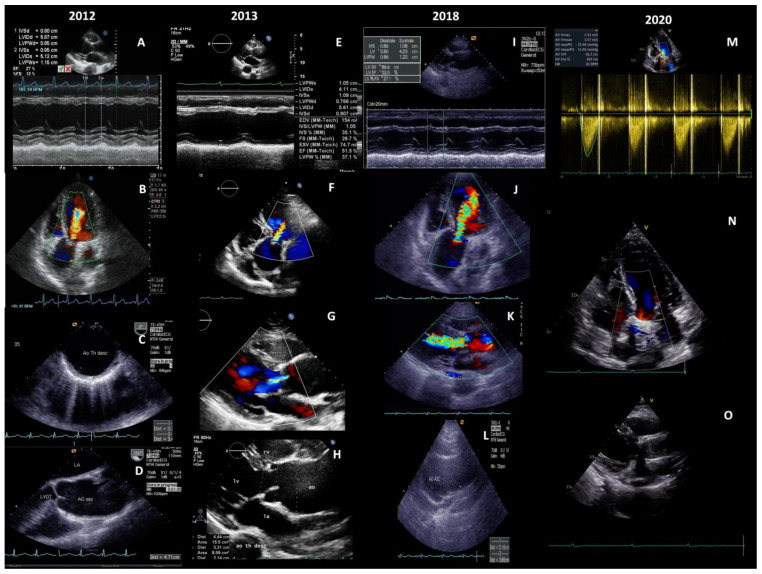
A 26-year-old female was diagnosed with an advanced stage of Takayasu arteritis (TA) in 2012. TA is a rare, large vessel vasculitis that involves aorta, its major branches, and occasionally pulmonary arteries [1]. At the time of diagnosis, she presented with inflammatory syndrome, chest and back pain, congestive heart failure (CHF) with left ventricular ejection fraction (LVEF) 27% (**A**), aortic regurgitation (AR) 2+ (**B**), aortic wall inflammation (**C**), and ascending aorta aneurysm (AAA) 4.7 cm (**D**) (Supplementary echo videos and figures: Chest X Ray, ECG, AAA wall, available at Folder 2012). Patients with TA can present with constitutional features (fever, malaise, anorexia, and weight loss), or various symptoms and signs caused by morphological changes in the vessels affected by the inflammatory process: bruits, absent or diminished pulses, reduced or asymmetric blood pressure, claudication, ishaemic/congestive heart disease or neurological symptoms [2]. Based on clinical features and typical angiographic findings, the patient fulfilled the 1990 American College of Rheumatology (ACR) classification criteria for TA [3]. Corticosteroids and immunosuppressives are considered the first line treatment in all patients with active TA, while biologics should be considered in case of relapsing or refractory disease [4]. After 6 months of immunosuppressive and conservative treatment for CHF, some clinical aspects of the disease temporarily improved: LVEF 51% (**E**), AR 1+ (**F**,**G**), AAA 4.4 cm (**H**) (Supplementary echo videos available at Folder 2013). Despite the normal CRP and ESR, clinical parameters of heart function continued to gradually deteriorate from 2015, and during 2018, AR of 3+ was found on echocardiography (**J**,**K**), with AAA reaching diameter 5.5 cm (**L**) (Supplementary echo videos available at Folder 2018). Considering further LV enlargement (**I**), a progression of AR and a significant increase of AAA**,** surgery was suggested. The patient has had an uneventful recovery during the postoperative course with no complications at one year follow-up. Normal LV diameter, LVEF 67%, a trace of AR (**M**,**N**), and the aortic graft 3.0 cm in diameter at the position of the ascending aorta, were seen on the last echocardiography (**O**) (Supplementary echo videos available at Folder 2020).

**Figure 2:**
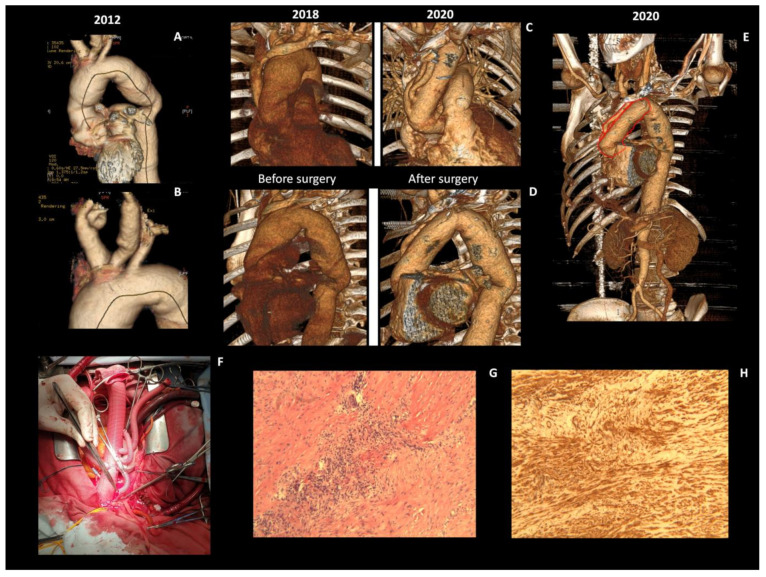
In August 2019, aortic valve, root and the ascending aorta (marked by the red line in (**E**)) replacement (Bentall procedure) with total arch reconstruction was performed (**C**,**D**,**E**). In addition, replacement of supra-aortic branches was done (four-branched dacron graft) (**F**) due to the multiple aneurisms and significant stenoses (**A**,**B**) clinically presented with recurrent neurological symptoms. It is well known that surgical treatment may improve the outcomes of patients with moderate-to-severe AR due to TA [5]. Histological examination of the aortic wall revealed a segmental fibrointimal proliferation, focal mononuclear cell infiltration (CD4^+^ and CD8^+^ lymphocytes, plasma cells and macrophages) in the outer thirds of the media (H&E) (**G**), smooth muscle cell disorganization by using immunohistochemistry analysis of α-smooth muscle actin (SMA) expression (**H**), and an adventitial fibrosis. Histological examination may be of a particular diagnostic value in TA, however biopsy of medium- to large-sized vessels is usually possible only at the time of vascular procedures or post-mortem [6]. Open surgery remains a treatment of choice for TA patients with moderate-to-severe AR and with AAA. However, surgical intervention should be considered in patients with clinically inactive disease at the time of intervention and must be led by an experienced multi-disciplinary team [7].

## Data Availability

The data presented in this study are available upon a reasonable request, from the corresponding author.

## Supplementary Materials

The following are available online at https://www.mdpi.com/2075-4418/11/2/216/s1, Figures: Chest X Ray, ECG, AAA wall at Folder 2012; Videos: echocardiography and Doppler ultrasound videos available at Folders 2012, 2013, 2018, 2020.
